# How do authors want to use AI for review? A survey to assess the perception of scientists who received both AI and human reviews of their manuscripts

**DOI:** 10.1038/s44319-026-00725-4

**Published:** 2026-02-26

**Authors:** Thomas Lemberger, Niv Samuel Mastboim, Oded Rechavi

**Affiliations:** 1 EMBO, Heidelberg, Germany; 2 qed Science, Tel Aviv, Israel; 3 School of Neurobiology, Biochemistry and Biophysics, Wise Faculty of Life Sciences and Sagol School of Neuroscience, Tel Aviv University, Tel Aviv, Israel

**Keywords:** Science Policy & Publishing

## Abstract

A survey of researchers who compared AI-generated scientific reviews with journal-agnostic human peer review reveals that they overwhelmingly prefer using AI as a self-checking tool before submission rather than as a replacement for human reviewers. It encourages an “author-centric” model in which AI helps researchers improve their manuscripts before they are reviewed by their peers.

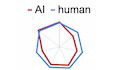

AI is advancing at a rapid pace: the reasoning capabilities of Large Language Models (LLMs) are improving; multimodality enables them to simultaneously analyze text, images, and other sources of data, and access to web resources is increasing their ability to retrieve information, access recent knowledge, and self-correct with autonomous fact-checking. Vigorous efforts are also underway to overcome fundamental limitations of auto-regressive LLMs, such as new ways to learn causal “world models”, to improve understanding of scientific concepts and AI model robustness (Richens and Everitt, 2024).

Along with these expanded capabilities of AI systems, applications dedicated to critically analyze and review scientific papers have become available as part of research projects (Bougie and Watanabe, 2024; D’Arcy et al, 2024), commercial platforms (Nature Research Assistant, https://natureresearchassistant.com; AlchemistReview, https://www.hum.works/review), specialized startups (qed, https://www.qedscience.com, Reviewer3, https://reviewer3.com) or even as self-described ‘weekend projects’ by prominent AI engineers (Andrew Ng’s agentic reviewing AI, https://paperreview.ai).

A major incentive of these efforts is to address several problems and bottlenecks of human-based peer-review (Mann et al, 2025). It is a time-consuming activity that is carried out by already overcommitted researchers and authors often have to wait for weeks or months for a decision on their manuscript. Finding reviewers with suitable expertise who are willing to evaluate manuscripts is increasingly challenging for journal editors. Moreover, human evaluation can be variable, affected by individual emotions, politics, and biases that are difficult to measure.

Nonetheless, the critical evaluation a scientific work along with advice on how to improve it is a fundamental aspect of the scientific process. Will AI be able to help mitigate these issues? Could it accelerate the publishing process while upholding quality standards and controlling for biases? Could it free up time for reviewers and decongest a system that is reaching saturation, helping scientists to focus on insight rather than checks and gatekeeping?

“… the critical evaluation a scientific work along with advice on how to improve it is a fundamental aspect of the scientific process.”

When it comes to the use of AI in publishing and peer review in particular, many fear its encroachment into domains once reserved for human judgment. What if machines are able to replace critical analysis of scientific claims, something that scientists greatly treasure and that is a crucial element of the scientific enterprise? What if AI carries over or even amplifies bias, reduces accountability, hallucinates, or becomes a tool for gaming the system? A recent commentary paper cautions that, when researchers relinquish scientific judgment to machines, we risk losing something essential about how science evolves: its human, critical core (Bergstrom and Bak-Coleman, 2025). In addition, authors might fear that AI will be a tougher critique than humans, as it could, in theory, find any small problem a paper might have, no matter how tiny.

AI is, however, here to stay. From writing papers to designing experiments and reviewing scientific results, its use is already widespread (Lemberger, 2024). It is therefore essential to engage with it carefully and constructively to develop the best use cases that benefit the scientific community. To start with, it is important to assess the attitudes of the main beneficiaries of AI-assisted peer review, namely, authors.

“… it is important to assess the attitudes of the main beneficiaries of AI-assisted peer review, namely authors.”

## Testing the potential: an experiment

To this end, we conducted a survey that involved side-by-side human peer review and AI scientific review: the comments by an AI were not compared to the comments received from individual human reviewers but instead to the combined feedback by multiple human peer reviewers, typically 3. In the text below, we will strictly reserve the term “*peer review*” for the human activity, whereas we will use “*AI (scientific) reviews*” for analyses produced by machines.

We invited a cohort of authors whose manuscripts had been evaluated by the preprint peer review platform Review Commons. This platform, managed by one of the authors (TL), runs a journal-agnostic and transparent peer-review process on submitted preprints, without making any post-review editorial reject/accept decisions. It thus provides a suitable test set for comparison with an AI review platform that also analyses papers without reference to a journal’s scope or editorial selection criteria.

Participating authors were provided with the AI scientific review produced by the qed platform, which was recently founded by two of the authors (NSM and OR; the version of qed used in this work was v1.0), as well as the human peer reviews they had obtained from Review Commons for comparison. To exclude the possibility that the AI platform would have access to the Review Commons reviews, we only selected papers whose reviews were not yet public at the cutoff date for the platform’s training (Oct 26, 2024). The fact that the AI reviews were provided retrospectively for papers that had already been peer-reviewed also reassured authors that the experimental AI reviews would not influence the fate of their manuscript.

The side-by-side comparison of the AI-generated review with a prior human journal-agnostic review was a good opportunity to ask questions about the strengths and weaknesses of both forms across multiple “quality features”. We were also interested in exploring the best use cases for AI reviews: should authors, reviewers or editors use these tools? Should preprints be reviewed by AI at scale? Even more provocatively: would authors consider autonomous AI-operated journals?

**Figure 1:**
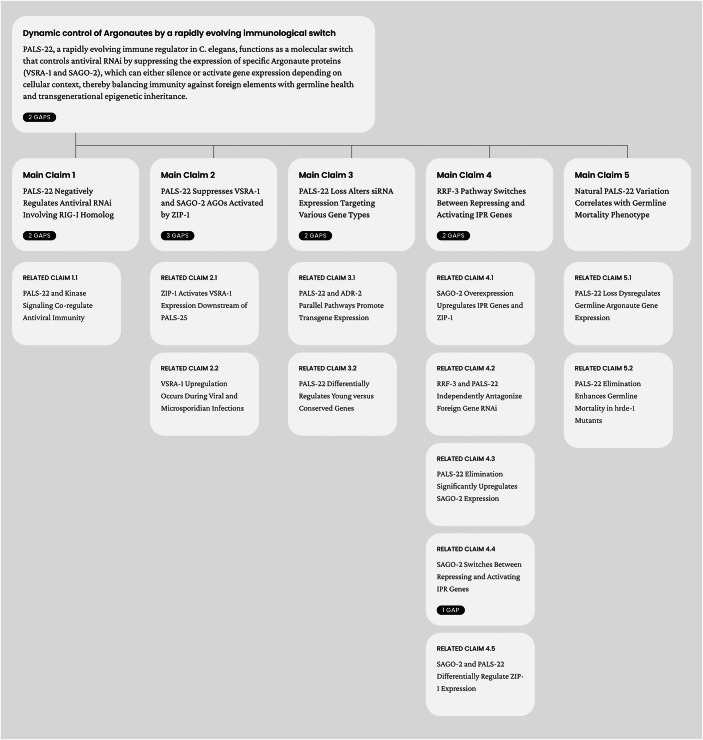
qed report logical claim tree. The platform identifies logical gaps, highlights inconsistencies and suggests possible solutions. While more advanced versions were released since, the version of the qed platform used in this work was v1.0. The report’s central thesis (top node) is decomposed into main claims and their supporting related claims, linked to show dependency structure. Black badges on each claim card indicate the number of identified gaps for that specific claim.

The qed AI agent analyzes the content of a paper to identify its major scientific claims and spot potential gaps in supporting these claims. When gaps are identified, the agent provides suggestions for addressing the issue, either through additional experimentation or by textual changes to “tone down” the respective claims. To improve the clarity of the analysis, the qed platform displays the review as a hierarchical “claim tree”, and “gaps”, if any, for each specific claim (Fig. 1). The format is intentionally different from classical human-written referee reports to improve clarity but also to make it immediately apparent that the review is generated by a machine and not by a human.

The responses to the experiment were both surprising and instructive. First, we found that our cohort is genuinely curious about using AI in scientific review: from 408 authors contacted, 181 (44%)% expressed interest in participating, and 126 (31%) engaged with at least part of the survey. This level of engagement was notable and suggests a strong interest. Given the small size and focused nature of the cohort, the results are interpreted only qualitatively to identify trends without seeking more quantitative insights or overgeneralizing to the larger life-science community. By design, the study is a side-by-side comparison of human peer review and AI review. Authors were therefore not blind to the origin of the review. The results can therefore be affected by biases toward or against the use of AI in research.

We first asked authors to rate the AI and human reviews across seven “quality features” to ask respondents whether the reviews were:

1. **Critical**: identifies the most relevant gaps accurately
2. **Detailed**: offers a good coverage of all aspects
3. **Constructive**: provides actionable suggestions
4. **Insightful**: deeply understands the goals and the relevance of the research
5. **Evidence-based**: supports critiques with reference to data, figures, content of the paper and literature citations
6. **Clear**: written and presented in a clear way
7. **Polite**: uses a professional and neutral tone.

**Figure 2:**
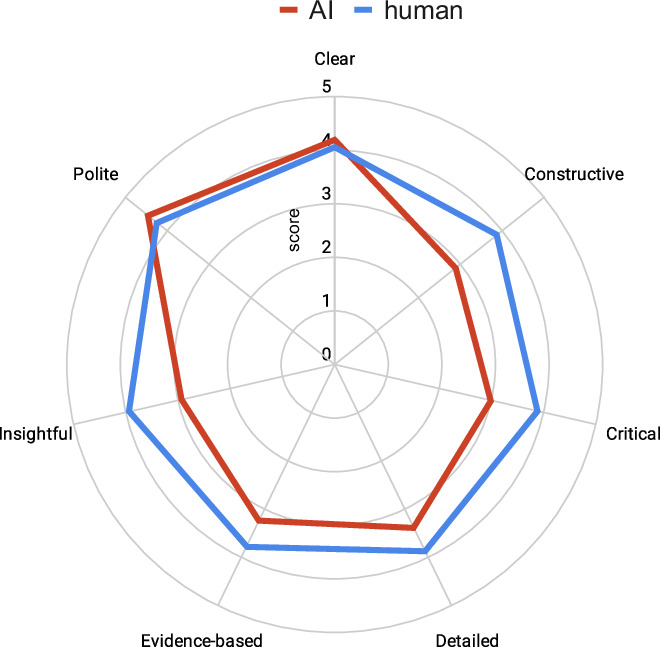
Perceived quality of qed AI vs. human peer reviews across seven review features. Spider plot showing mean author ratings (0–5 scale; higher is better; *n* = 109) for AI-generated reviews (red) and human peer reviews (blue) across seven quality features: critical, detailed, constructive, insightful, evidence-based, clear, and polite.

The results show that, overall, human peer review was rated higher for scientific understanding and insights, with the exception of clarity and politeness, where AI and humans were largely on par (Fig. 2). Many respondents indicated they were impressed that machines can already reach a level of understanding sufficient to provide helpful feedback on scientific content.

**Figure 3:**
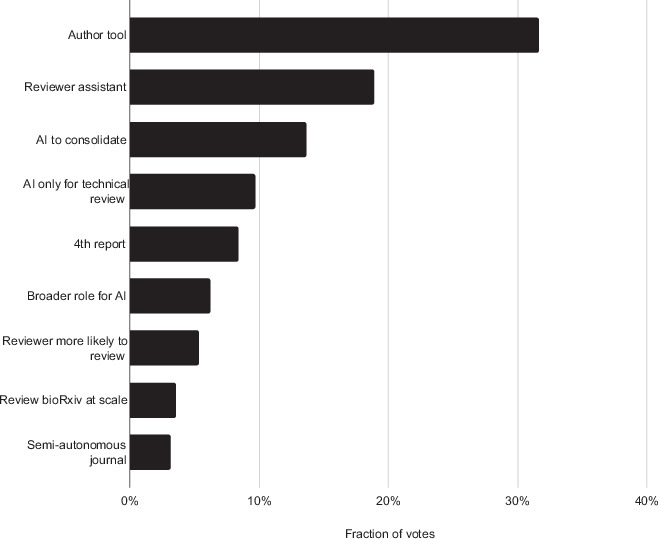
Potential use cases of AI in scientific review. Participants (*n* = 98) voted to prioritize the most promising use cases, with each participant allowed to vote for up to three different options. The bar values represent the fraction of votes received by each use case (votes for that use case divided by the total number of votes, *N* = 228, cast across all use cases).

Second, we asked respondents to vote for various potential use cases for AI-based scientific reviews (Fig. 3). Respondents could vote for up to three of the following propositions:

- **Authoring tool (self check):** *“As an author, I want to use AI to review my work myself, so I can improve it before submission or before posting the preprint.”*
- **Reviewers will be more likely to review:** *“As a reviewer, I am more likely to accept reviewing if the authors have pre-reviewed and revised their work with AI.”*
- **Additional “fourth” report:** *“Review Commons and journals should include an AI scientific review as a ‘fourth report’, to provide a complementary evaluation.”*
- **Semi-autonomous journal:** *“I would be happy to submit to a very fast journal that only uses AI scientific reviews, provided an editor maintains oversight of the quality and relevance of the AI reviews.”*
- **Reviewing assistant:** *“As a reviewer, I would like to use AI review to cross-check my own report, so that I can deliver a better peer review.”*
- **Review every preprint**: *“Every bio/medRxiv preprint should be reviewed by AI, and the reports made publicly available.”*
- **AI for technical review only:** *“AI should be used only for specialized technical aspects of a paper, not for in-depth evaluation of a scientific work.”*
- **Broader role for AI:** *“AI has the potential to assume a broader role in the review process, gradually replacing many aspects of human review”*.
- **AI only to consolidate human review:** *“AI should primarily be used to consolidate and harmonize the available human peer reviews.”*

The clear winning option is the use of AI review by authors to self-check their manuscript prior to submission (Fig. 3). The second most popular option was the use of AI as a reviewing assistant. There was, in contrast, strikingly little appetite for journals that would rely mainly on AI reviews, even if moderated by human editorial oversight – we acknowledge that this response in particular could differ depending on the target journal. Similarly, the possibility of reviewing all preprints at scale was not perceived as an attractive option to this cohort. Future studies are required to assess whether this reflects a fear that AI is too critical or unjust, and whether further improving the quality and insightfulness of AI reviews could change this perception.

“The clear winning option is the use of AI review by authors to self-check their manuscript prior to submission.”

In the free-text feedback from respondents, the perception of AI’s role in the review process remained somewhat mixed. Some authors expressed surprise that a machine could “understand” a scientific paper at this level: “*AI was surprisingly good in reading the manuscript, understanding the point of it, and identifying gaps*.” Some even felt that it could be superior to humans: “*It was pretty spot on. Much more to the point than the human reviewers, which tend to pick up points that are not really relevant*”. Many of the survey’s participants felt that AI review requires human oversight and should not replace human peer review: “*AI reviews are an interesting complement, but currently the way the author addresses these comments should remain the job of an editor (human) not AI*”. Broader risks were also noted, such that AI review could lead “*to a loss of diversity and genuine criticism based on each person’s experience and way of thinking*” and “*a progressive homogenization of the type or review reports*”.

## An author-centric tool, not a gatekeeper

The results suggest that our cohort of researchers is curious about AI review and cautiously open to using it as an assistive tool. As one of the respondents expressed: “*I would love to use AI to review my work myself and alert me to the stock critiques (standard, generic, slightly annoying criticisms) that I might get from human reviewers*.” While transparency is crucial in science, authors may not always want their initial paper scrutinized for every small detail in public. Some mistakes might be rather embarrassing and are better caught before the paper is publicly available. When you dress up for an event, it can be a good idea to first ask your partner or close friends, “*How do I look? Does this fit the dress code?*” This initial feedback, even if direct and uncompromising, can save you from the greater embarrassment of showing up without pants or, less dramatically, in a bowtie when everyone else is wearing a T-shirt. More seriously, pre-checking scientific work with AI could allow researchers to share a public version of the paper faster and more confidently, even if it’s not perfect. Human evaluation at later stages will still be important, and taking into account the constructive criticisms from peers will remain a core part of the scientific process.

Based on the feedback from our survey, we believe AI can be adopted to address some of the problems of scientific publishing mentioned above. While still imperfect, an AI agent offers unique strengths: it is extremely fast, and it won’t compete with you. It never gets tired, has infinite patience, and can read every figure and supplement, cross-check statistics, and, when properly designed, can provide consistent feedback. To reap its benefits, however, we should follow two guiding principles: Author-centeredness and Transparency.

AI reviews should help authors, not subjugate them. We envision a system where authors can refine early drafts, anticipate critical feedback, stimulate internal discussion, and engage more symmetrically with the review process before they submit their work to a journal or preprint server. We see AI feedback as an additional lens rather than a gatekeeper, akin to a “pre-flight check” to speed up the process and make it more efficient.

“AI reviews should help authors, not subjugate them.”

In addition, it should be clear that an AI scientific review is different from a human-written peer review. An AI is a machine, not a “peer”. Humans may—for now—have superior critical thinking and reasoning capabilities. The importance of the social fabric of science can also not be underestimated and human-to-human dialog, feedback and debate are an integral part of the scientific enterprise (Kuhn, 1962). It is thus essential that AI reviews are delivered in a transparent manner that makes it unmistakable that they were generated by a machine.

In this context, it is intriguing that the second-most-voted use case is for reviewers to use AI to cross-check and help them produce better referee reports. This scenario is currently a source of great concern in scientific publishing, especially as poor-quality, undeclared AI-generated reviews have begun to contaminate the peer-review process (Naddaf, 2025). Authors who receive such reviews feel dismayed and powerless – as two of the authors of this article, TL and OR, can testify. It will thus be necessary to mandate and enforce transparency, requiring the use of recognized or even certified dedicated tools, requiring reviewers to declare the use of AI tools, verifying their identity, and posting the reviews publicly so they can be screened and debunked if necessary.

## The future

For broad adoption and more systematic use by researchers, a crucial issue is measuring and documenting the ‘quality’ of AI reviews to compare their performance on an objective basis. Our seven ‘review quality features’ scoring may serve as a basis for developing multidimensional metrics to compare AI review platforms, but, as an interesting side effect, could also be used to compare human peer review across different services.

AI review is in its infancy. The feedback obtained in our experiment is already encouraging, and it is reasonable to assume that AI review systems will continue to improve over time. Policies grounded on a current snapshot of the state of AI, or those that bet on hypothetical, insurmountable difficulties in improving AI, are most likely to be misguided and rapidly obsolete. We also agree with Mann et al (2025) that a constructive evaluation of “LLM use in peer review should compare the current state of the art to the status quo, not yesterday’s models to an idealized human reviewer.” The future will tell how journals decide to integrate AI review in their workflow. We look forward to the next stage in the evolutionary process, which might give rise to a hybrid review model in which human and AI synergize. The aim should be to reduce reviewer burden, shorten decision timelines, and shift reviewer effort toward deeper insight rather than checking errors. The onus is on AI platforms to achieve and document a level of quality that will convince researchers and journals to incorporate AI review as a standard step in the research and publishing workflow.

“The aim should be to reduce reviewer burden, shorten decision timelines, and shift reviewer effort toward deeper insight rather than checking errors.”

However, the path is not without risks. AI systems could amplify biases in training data, misinterpret creative or speculative work, or miss nonconventional contributions. Transparent validation, clear error bounds, and “humans in the loop” are essential. Past and future analyses will hopefully provide more empirical grounding for a cautious, community-driven rollout (Manrai et al, 2025; Mann et al, 2025; Liang et al, 2024). Our author-centric pilot was motivated by the desire to engage with scientists as active users of AI (and we don’t want to be the technicians of the robot overlords!), as they are the best experts to shape, with a critical mind, how the technology should be used. Our results suggest that scientists are open to AI review, and thus give reason for guarded optimism.

## Supplementary information

Peer Review File
