# Supplementary material for: How do authors want to use AI for review? A survey to assess the perception of scientists who received both AI and human reviews of their manuscripts
Source: EMBO Rep. 2026 Feb 26;27(6):1388–92. doi: 10.1038/s44319-026-00725-4 (PMC13022281; doi:10.1038/s44319-026-00725-4)
Supplement: Supplementary file 1 — Peer Review File [file 44319_2026_725_MOESM1_ESM.pdf]

EMBO reports

How Do Authors Want To Use AI For Review?

Thomas Lemberger (MSB), Niv Mastboim, and Oded Rechavi

Corresponding author(s): Thomas Lemberger (MSB) (thomas.lemberger@embo.org)

Review Timeline:

Submission Date:	8th Feb 26
Accepted:	10th Feb 26

Editor: Holger Breithaupt

Transaction Report: This article was editorially reviewed.
